# The *Aspergillus nidulans* transcription factor SclB governs the transition from vegetative to asexual development

**DOI:** 10.1128/mbio.03488-25

**Published:** 2026-02-25

**Authors:** Emmanouil Bastakis, Rebekka Harting, Alexandra Scheel, Tanja Lienard, Christoph Sasse, Merle Aden, Gabriele Heinrich, Verena Grosse, Nicole Scheiter, Gerhard H. Braus

**Affiliations:** 1 Department of Molecular Microbiology and Genetics, Institute for Microbiology and Genetics, University of Göttingen, Göttingen, Germany; 2 Department of Prosthodontics, University Medical Center, Göttingen, Germany; The Lundquist Institute, Torrance, California, USA

**Keywords:** transcription factors, asexual development, *Aspergillus nidulans*, *Verticillium dahliae*, *sclB*, *brlA*, *ppoC*, *veA*, *velB*

## Abstract

Asexual reproduction in filamentous fungi is a common, efficient, and fast differentiation process for producing large numbers of asexual spores (conidia), which can be distributed through the air to colonize new environments. The whole process is tightly controlled by specific regulatory proteins. Among the major regulators is the zinc cluster protein of SclB (sclerotia-like B), known to influence various aspects of asexual growth and secondary metabolism in *Aspergillus nidulans* as well as other filamentous fungi. Two different growth conditions of *A. nidulans* were compared to obtain a mechanistic overview of the role of SclB, mainly during the transition of the fungus from vegetative to asexual growth. Chromatin immunoprecipitation (IP) was coupled with next-generation sequencing (ChIP-seq) and combined with transcriptomic analyses (RNA-seq). SclB coordinates this developmental shift mainly by controlling the expression of genes encoding a few, however, prominent regulators of conidiation. These include the transcription factors BrlA, VelB, and SclB and the pheromone oxygenase PpoC. Association of SclB to promoter regions requires the newly identified putative SclB response element with a nine-base-pair DNA motif. Scl2 is the corresponding protein in the fungal plant pathogen *Verticillium dahliae* and partially complements the *A. nidulans* Δ*sclB* asexual deficiency. This supports a partial functional complementation of this regulator among different fungal species. In summary, SclB coordinates the transition from vegetative growth to asexual reproduction in *A. nidulans* through *in vivo* transcriptional control over genes coding for established players of conidia formation.

**IMPORTANCE:**

Fungi constantly adapt to environmental changes in their various habitats. Asexual spore formation allows for quickly leaving an unfriendly habitat through dispersal into the air. The asexual developmental program of fungi enables the production of a large number of spores in a short period of time and in an energetically efficient manner. The SclB transcription factor is a key regulator of asexual growth and secondary metabolism in numerous fungal species. The mechanism through which SclB orchestrates the transition of the filamentous fungus *Aspergillus nidulans* from vegetative to asexual growth was revealed. This regulator directly *in vivo* controls itself as well as the expression of master genes for the asexual program, such as *brlA* for transcriptional control or *ppoC* for pheromone production. This study enhances the molecular understanding of how fungal asexual differentiation is initiated and coordinated, which supports the development of better strategies to control fungal pathogens, improving human health, safety, and crop management.

## INTRODUCTION

Conidia are the final products of the asexual developmental program in many filamentous fungi. From the stage of mycelia (undifferentiated hyphae) till conidia production, the fungus is undertaking a variety of structural and conformational changes (1). These changes are controlled by regulatory transcription factors (TFs), which constitute the end-line receivers of signals generated from external environmental inputs or internal processes (2). TFs convert these signals into specific gene expression changes, leading ultimately to the production of proteins necessary for the growth under specific conditions.

The asexual developmental program consists of multiple transcriptional regulatory levels and interactions. The central player for the onset of asexual sporulation is the transcriptional activator BrlA (bristle A) (3). SfgA (suppressor of *fluG*), VosA (viability of spores A), and NsdD (never in sexual development D) are TFs that occupy the *brlA* promoter and repress its expression (3–5). Under asexual conditions, the FluG (fluffy G) regulator attenuates the effects of these three repressors. That leaves the promoter of *brlA* free to interact with the so-called FlbB (fluffy low brlA), FlbC, and FlbD activator proteins (6, 7). The regulators, contributing to the induction of *brlA*, are called upstream developmental activators (6). BrlA controls genes necessary for the completion of sporulation, for example, the formation of sporogenus phialides (*abaA*) (8) and the structuring and maturation of conidia (*wetA*) (9). AbaA (abacus A) controls the expression of *brlA*, *abaA,* and *wetA* (*wet-white A*), thereby influencing the progress and completion of asexual development (8). BrlA, AbaA, and WetA are key control elements of the so-called central developmental pathway (CDP) for the initiation of sporogenesis, maturation, and finally the survival of conidia as asexual spores to be released into the air (10).

The velvet domain family is a fungal-specific group of transcriptional regulators with central roles in development, which link differentiation to the appropriate specific secondary metabolism in *Aspergillus nidulans,* as well as in other fungi (1). The velvet DNA-binding domain associates *in vivo* with promoter regions and has the same fold as mammalian NF-kappa (11). The ability of velvet domain proteins to form homodimer or heterodimer provides a complex regulatory system of distinct homodimer or heterodimer (1, 12). VelB is an inducer of asexual growth, since its deletion strain produces fewer conidia and shows reduced expression of the asexual regulators *brlA*, *abaA,* and *vosA* (13). VeA, VelC, and VosA have various prominent roles during sexual development but also operate as repressors of asexual growth (13–15).

Pheromones are essential for the reproduction of filamentous fungi. In *A. nidulans,* the proportion of three psi (precocious sexual inducer) factors, psiA, psiB, and psiC, defines the shift between sexual and asexual development (1). Specifically, the dioxygenase PpoC (psi factor producing oxygenase C), in charge of the synthesis of psiBβ, is an inducer of conidiation (1, 16).

Fungal zinc cluster proteins include a great number of TFs characterized by a cysteine residue linked to two zinc atoms (17). The *A. nidulans* sclerotia-like B (SclB) zinc cluster protein has been associated with the regulation of asexual development and secondary metabolism, but also as a direct repressive target of VosA (11, 18). More recently, SclB was also shown to be involved in the coordination of secondary metabolism in fungal-fungal cocultivation (19). The implication of the protein in asexual development was originally shown in *Aspergillus niger* (20), where in a UV mutagenesis screen, two separate mutants were isolated, *scl-1* and *scl-2*. Both showed severe defects regarding asexual sporulation alongside the formation of sclerotia-like structures during growth on plates.

Here, we show that *A. nidulans* SclB is specifically important for the transition from vegetative to asexual growth. It directly affects the expression of genes coding for master regulators of asexual growth, such as the TFs BrlA, VelB, and SclB, or the pheromone enzyme PpoC. *V. dahliae* Scl2 can partially rescue the strong phenotype of *A. nidulans* Δ*sclB*, which supports a conserved function of SclB among different fungal species.

## RESULTS

### The asexual master regulator SclB has distinct *in vivo* binding and gene expression profiles across the fungal genome during vegetative compared to asexual growth

During vegetative growth, SclB already controls the expression of genes coding for proteins with prominent roles during subsequent asexual development (18). Currently, it is unknown whether this regulation is based on direct *in vivo* association of SclB with the promoters of these genes or whether additional intermediate regulators are involved. ChIP-seq studies were performed in combination with a transcriptomic RNA-seq gene expression analysis to examine whether this transcriptional control is based on a direct SclB interaction.

**Fig 1:**
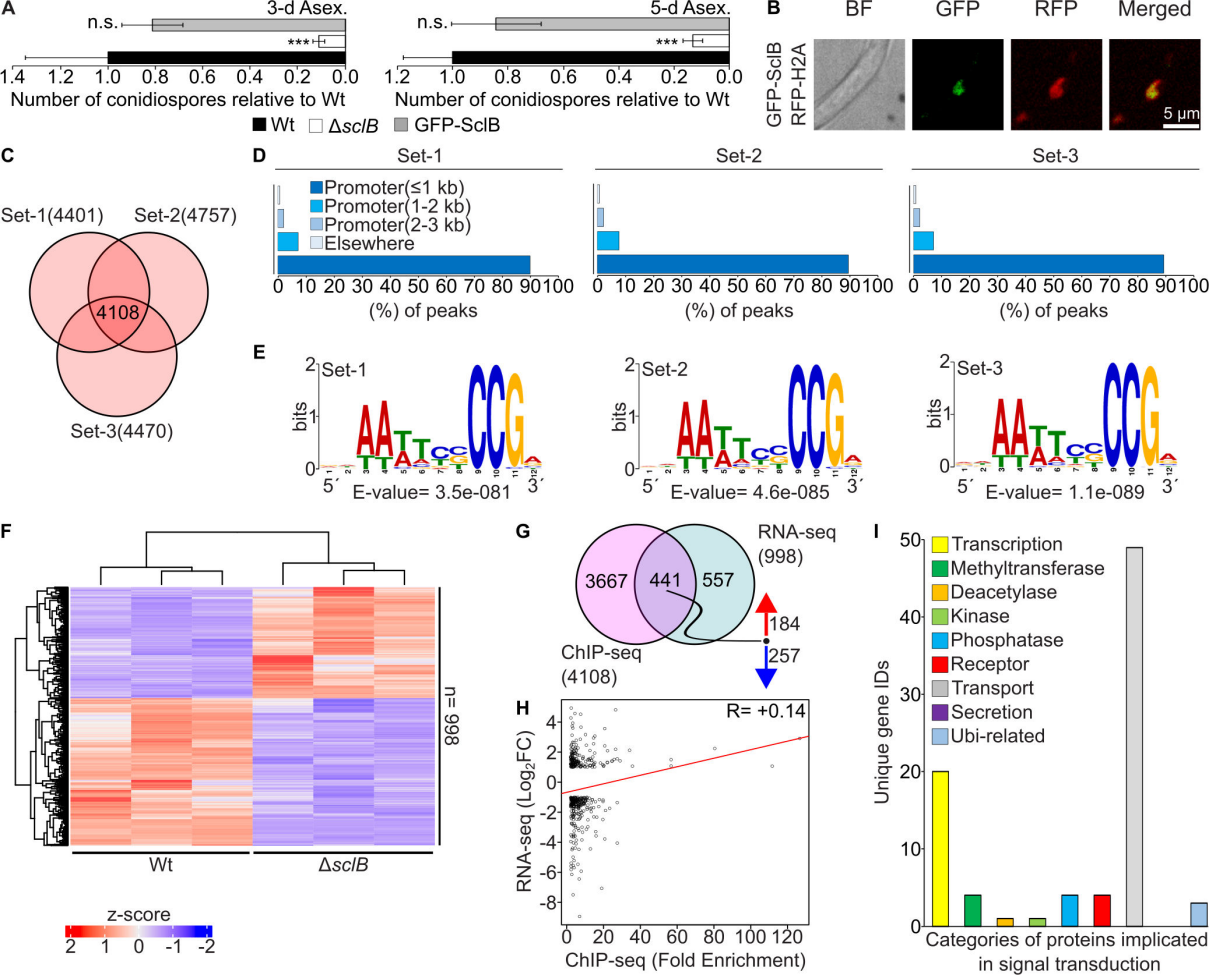
*In vivo* binding landscape of the GFP-SclB transcriptional regulator during vegetative growth of *A. nidulans*. (**A**) Bar plots presenting the quantification of asexual spores (conidia), for Wt, Δ*sclB,* and GFP-SclB strains after 3 days or 5 days of growth under Asex conditions. The total number of spores is normalized to the Wt, presenting averages and standard deviations of five independent replicates (plates) for each strain. A total of 30,000 spores was initially inoculated on each plate; spores were then spread all over the plate using glass beads. (**B**) Cellular *in vivo* localization of SclB, tagged by a GFP at its N-terminus. GFP-SclB is expressed under the native promoter of *sclB*. The same strain additionally carries a transgene expressing the universal nuclear marker H2A tagged with RFP (red fluorescent protein) at its N-terminus. For the confocal images, hyphae were grown under vegetative growth conditions. (**C**) The Venn diagram presents the overlap of three independent sets of ChIP-seq analysis for GFP-SclB versus Wt. ChIP-seq was performed with mycelia grown for 20 h under vegetative growth conditions (hereafter Veg). In sum, 4,108 unique gene locus IDs were found to be associated with peaks (cutoffs: *P* < 0.05 and F.E. ≥ 2.0) located in up to 3 kb promoter regions, discovered simultaneously in all three independent sets of the ChIP-seq analysis. (**D**) Bar diagrams depict the distribution of the statistically significant ChIP-seq peaks over different genetic elements for each of the three independent sets of analysis. For all three sets, approximately 90% of the identified peaks are located within promoter regions of genes up to 1 kb from the TSS. (**E**) Logos depict the top-ranked *de novo* motifs, as discovered by using the MEME-ChIP web tool. An input set of 150 sequences, each consisting of a length of 100 bp, was used for each independent set of analysis. All sequences were located below the summits of the top-ranked 150 ChIP-seq peaks for each set. The summits of all peaks were located up to 3 kb promoter regions. (**F**) Heatmap shows the differential expression of genes (as z-score values) from fungal mRNAs of Δ*sclB* and Wt strains, derived from mycelia grown under Veg conditions. The total number of genes found to be differentially expressed (n) under the cutoffs reflects *P* < 0.05 and −1 ≥ log_2_fold change (FC) ≥ 1. The RNA-seq was performed with three independent biological replicates of Δ*sclB* and Wt strains, respectively. (**G**) Venn diagram showing the overlap among the highly reproducible target genes from the ChIP-seq of GFP-SclB (**C**) with the differentially expressed genes (DEGs) from the RNA-seq of Δ*sclB* (**F**). Both experiments were performed with mycelia grown under Veg growth conditions. The corresponding cutoffs for the ChIP-seq were: *P* < 0.05 and F.E. ≥ 2.0 and for the RNA-seq: *P* < 0.05 and −1 ≥ log_2_FC ≥ 1. The numbers beside the red and blue arrows indicate either upregulated or downregulated genes from the total genes of the overlap. This set of 441 locus IDs reflects the direct *in vivo* targets of GFP-SclB for mycelia grown under Veg conditions. (**H**) Scatter plot showing the Pearson correlation coefficient between the peak’s F.E. from the ChIP-seq in panel **C** with the gene expression as of Log_2_FC from the RNA-seq in panel **F**, under the same Veg growth. For this plot, only the 441 direct target genes found in the overlap of ChIP-seq with the RNA-seq in panel **G** were used. The *R* = +0.14 inside the plot indicates the positive correlation between the specific NGS data sets tested. (**I**) Bar chart depicting the number of genes from the overlap (441 gene locus IDs) between the ChIP-seq and RNA-seq (as shown in panel **G**) belonging to categories of genes encoding proteins, known to be implicated in signal transduction processes. The number for each category was defined after performing a manual search of the set of 441 gene loci. n.s.: not significant.

For generating the *in vivo* binding profile of SclB, the *A. nidulans* green fluorescence protein (GFP)-SclB strain constructed by Thieme et al. (18) was used. The phenotypic appearance in terms of colony formation was found to be identical with that of the Wt strain. The GFP-tagged protein (GFP-SclB) was detected in Western experiments using α-GFP antibody in extracts from vegetatively grown hyphae (18). We further confirmed full functionality of this strain, prior to its use in the *in vivo* binding studies, by quantifying the total number of conidia after 3 days and 5 days of asexual growth (Fig. 1A), and confirmed its nuclear localization *in vivo* (Fig. 1B). Subsequently, ChIP-seqs were performed with mycelia of the GFP-SclB strain grown either vegetatively for 20 h (hereafter Veg) or just afterward were transferred to solid medium and grown in asexual growth conditions for another 3 h (hereafter Asex). Among the different biological replicates for each ChIP-seq, around 4,000 unique gene locus IDs were identified to be targeted by SclB in both conditions (Fig. 1C; Fig. S1A). In both experiments, SclB was associated *in vivo* with promoter regions spanning up to 3 kb from the transcriptional start site (TSS). Regions of the genome with a statistically significant (*P* < 0.05) enrichment (fold enrichment [F.E.] ≥ 2) of mapped read sequences, as derived from GFP-SclB samples versus wild type (hereafter Wt) (negative control/background signal), are named as peaks and indicated positions where SclB association with these regions occurred.

Subsequently, it was examined how the unique peaks are distributed over different genetic elements along the fungal genome. It was found that close to 90% of the identified peaks, for vegetative or asexual ChIP-seq, were located in promoter regions of genes that are extended up to 1 kb from the TSS (Fig. 1D; Fig. S1B). The DNA-binding motif, through which SclB association occurs, was elusive. Sequences of 100 bp located directly below the summit of the 150 top-scored peaks (based on the F.E.) were used to discover this motif for each independent set of the ChIP-seq analysis. A nine-base-pair DNA motif (consensus: 5′-AATTCCCCG-3′) with consistent composition among the different sets was discovered (Fig. 1E; Fig. S1C). This motif is recognized by SclB and was accordingly named putative SclB response element (PSRE).

The *in vivo* association of a TF does not always mean immediate transcriptional regulation of the nearby genes, because coregulation with other regulators is required, or the change in the expression of gene(s) takes place at a different developmental point or condition (21). Hence, the binding events of SclB were identified, which are truly leading to direct transcriptional regulation of genes located in close proximity to the corresponding peaks. Therefore, genome-wide gene expression analyses by RNA-seq assays were performed under the same vegetative and asexual growth-inducing conditions (Veg/Asex) as before, by comparing Δ*sclB* and Wt strains. The principal component analysis, among the different replicates of each of the sample groups, showed a clear separation and clustering between both RNA-seqs (Fig. S2). A total number of 998 genes for the Veg and 609 for the Asex were found to be differentially expressed (cutoffs: *P* < 0.05 and −1 ≥ log_2_FC ≥ 1) when comparing Δ*sclB* and Wt samples (Fig. 1F; Fig. S1D). Moreover, the visual representation of these DEGs, as indicated in the corresponding heatmaps, illustrated a distinct expression profile (presented as z-score values) of these genes during the tested growth conditions.

We then set to define, by overlapping these two data sets, the so-called direct *in vivo* target genes of SclB. A set of 441 unique gene locus IDs was found to be simultaneously differentially expressed and targeted by GFP-SclB at their promoter regions of up to 3 kb from the TSS *in vivo* (Fig. 1G) for vegetatively grown mycelia. However, the number of the direct SclB target genes was reduced almost in half (241) for mycelia induced during asexual development (Fig. S1E). Comparisons between SclB upregulated and downregulated target genes revealed that SclB mostly operates as an inducer during Veg and as a repressor during Asex growth conditions, respectively. A calculated Pearson correlation coefficient among the corresponding ChIP-seqs and RNA-seqs revealed a clear linear positive correlation (+0.14) for vegetative (Fig. 1H), in contrast to a slightly negative correlation (−0.06) during asexual growth conditions, respectively (Fig. S1F).

SclB controls various gene regulatory networks (18). The sets of 441 (Veg) and 241 (Asex) direct target genes of SclB were manually searched for candidates encoding proteins with functions in signal transduction. Under both conditions, the TF affected two overrepresented categories, which correspond to genes encoding proteins associated either with cellular transport or related to transcription (Fig. 1I; Fig. S1G). Further analyses with a focus on biological processes of the same sets for statistically significant enrichment of Gene Ontology (GO) revealed that most other overrepresented categories were associated with processes related to fungal primary or secondary metabolism (Fig. S3A and B).

**Fig 2:**
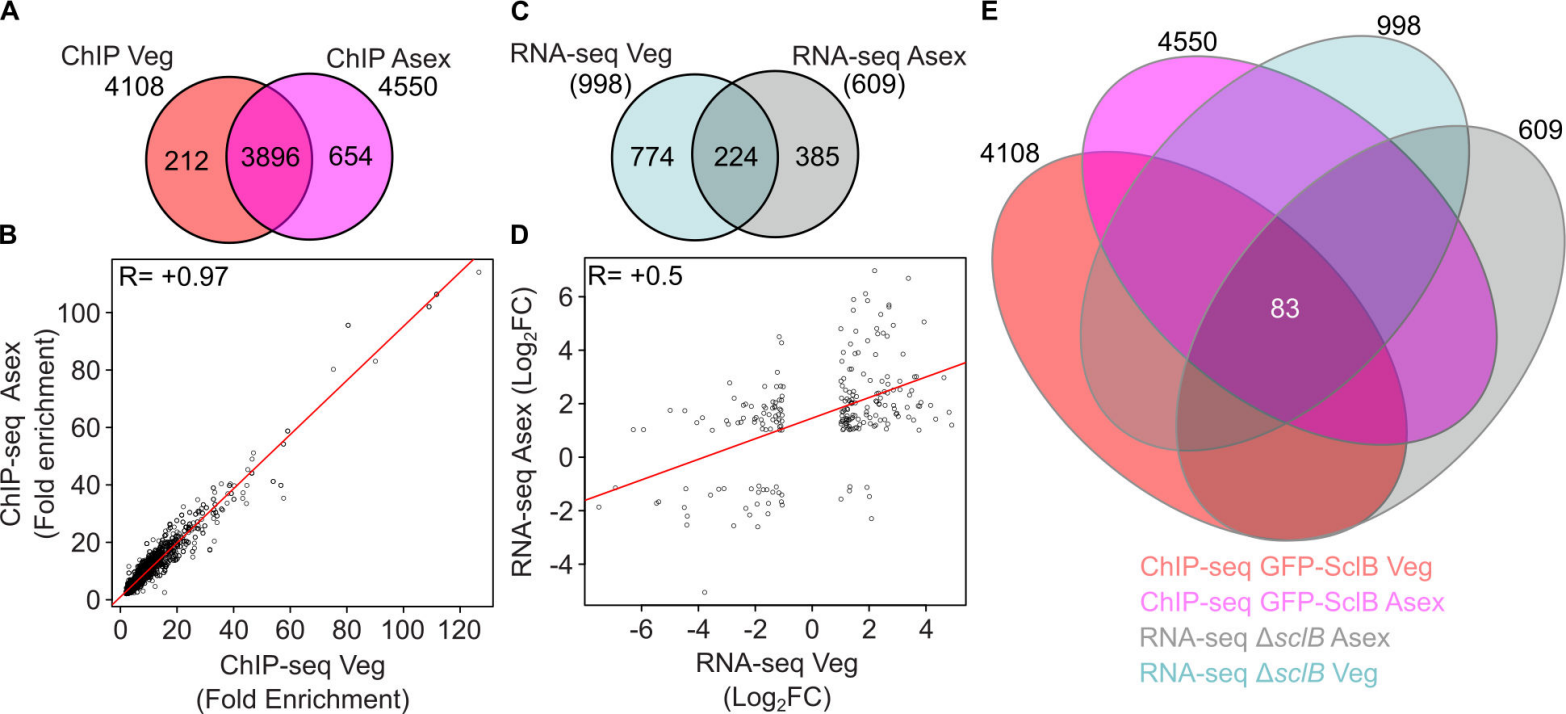
SclB directly targets 83 *A. nidulans* genes during different parts of the life cycle. (**A**) A Venn diagram illustrates the overlap of 3,896 gene locus IDs between the ChIP-seq experiments conducted under the two different growth conditions of Veg (as presented in Fig. 1C) and Asex (as presented in Fig. S1A) growth. For both of the ChIP-seqs, a prior filtration was applied for the identified peaks under the cutoffs: *P* < 0.05 and F.E. ≥ 2.0, the peak must be located up to 3 kb upstream from the TSS of every gene locus. (**B**) Scatter plot illustrating a Pearson correlation coefficient, between the F.E. of peaks derived from the ChIP-seq of the Veg-grown mycelia and the ChIP-seq with Asex-grown mycelia. For both ChIP-seq experiments, the following filtering conditions were applied: *P* < 0.05 and F.E. ≥ 2.0. For both ChIP-seq experiments, the set of locus IDs of genes was used that showed reproducible (in all three independent sets of each analysis) peaks to promoter regions up to 3 kb to their promoters. The *R* = +0.97 at the upper left corner of the plot shows the highly positive correlation among the compared NGS data sets that were examined. (**C**) Venn diagram depicting the overlap of gene locus IDs found to be differentially expressed under both conditions examined by RNA-seq (as presented in Fig. 1F; Fig. S1D). Mycelia derived from Δ*sclB* and Wt strains, under Veg and Asex growth conditions, correspondingly. Each RNA-seq consists of three biological replicates of the Δ*sclB* and another three biological replicates for the Wt strain. Both RNA-seq experiments were subjected to the cutoff: *P* < 0.05 and −1 ≥ log_2_FC ≥ 1. (**D**) Scatter plot presenting a Pearson correlation coefficient, among the expressions (log_2_FC) of genes found to be differentially expressed in the RNA-seq with the Veg grown mycelia and with the Asex grown mycelia. Both RNA-seq experiments were subjected to cutoffs: *P* < 0.05 and −1 ≥ log_2_FC ≥ 1. The *R* = +0.5 at the upper left corner of the scatter plot indicates a strong positive correlation among the DEGs of the different RNA-seq data sets. (**E**) Multi-Venn diagram highlighting a total number of 83 gene locus IDs found in both ChIP-seqs and both RNA-seqs performed under Veg and Asex growth conditions.

The *in vivo* binding profiles and the expression data from vegetative and asexual growth were further compared for overlaps and differences. The high positive Pearson correlation coefficient (+0.97) supports that the *in vivo* targets of SclB are largely maintained between vegetative and sexual growth (Fig. 2A and B). The two transcriptomic data sets further corroborate this finding with a positive (+0.5) correlation (Fig. 2D).

In contrast, the overlap of the differentially expressed genes (224 DEGs) between the two RNA-seqs was found to be severely reduced at roughly one-fifth from the Veg and one-third for the Asex data set, respectively (Fig. 2C). Genes, which are conserved direct targets of SclB under vegetative as well as asexual growth conditions, were identified by a Venn diagram between all four NGS data sets. A set of 83 genes could be identified as SclB target genes that remained associated with the TF under all tested conditions (Fig. 2E). The comparison of the expression patterns of these 83 genes revealed that 57 candidates maintained the same expression pattern during vegetative or asexual growth (Fig. S4). In contrast, 26 genes had an opposite expression profile after the transition to the asexual phase (Fig. S4). In sum, these results suggest that the *in vivo* affinity of SclB to promoters of a particular set of 83 genes (mostly related to metabolism, cellular transport, and transcription) is largely conserved during the shift from vegetative to asexual growth.

### SclB-mediated transcriptional control of *brlA*, *ppoC,* and *sclB* orchestrates the transition from fungal vegetative to asexual growth *in vivo*

SclB influences the expression of genes encoding major key regulators of asexual development (18) in mycelia grown either 24 h vegetatively or during the final stages of asexual development (24 h post-induction). The direct influence of SclB on the expression of core asexual regulators was examined, specifically during the transition from vegetative to asexual growth, by comparison with a list of established asexual regulators published by Krijgsheld et al. (22). This list consists of 35 genes, to which we added one more, *sclB* (AN0585) itself, published and characterized at a later time by Thieme et al. (18). The set of 36 genes of established asexual regulators (hereafter asexual MRs) was used for the comparisons.

**Fig 3:**
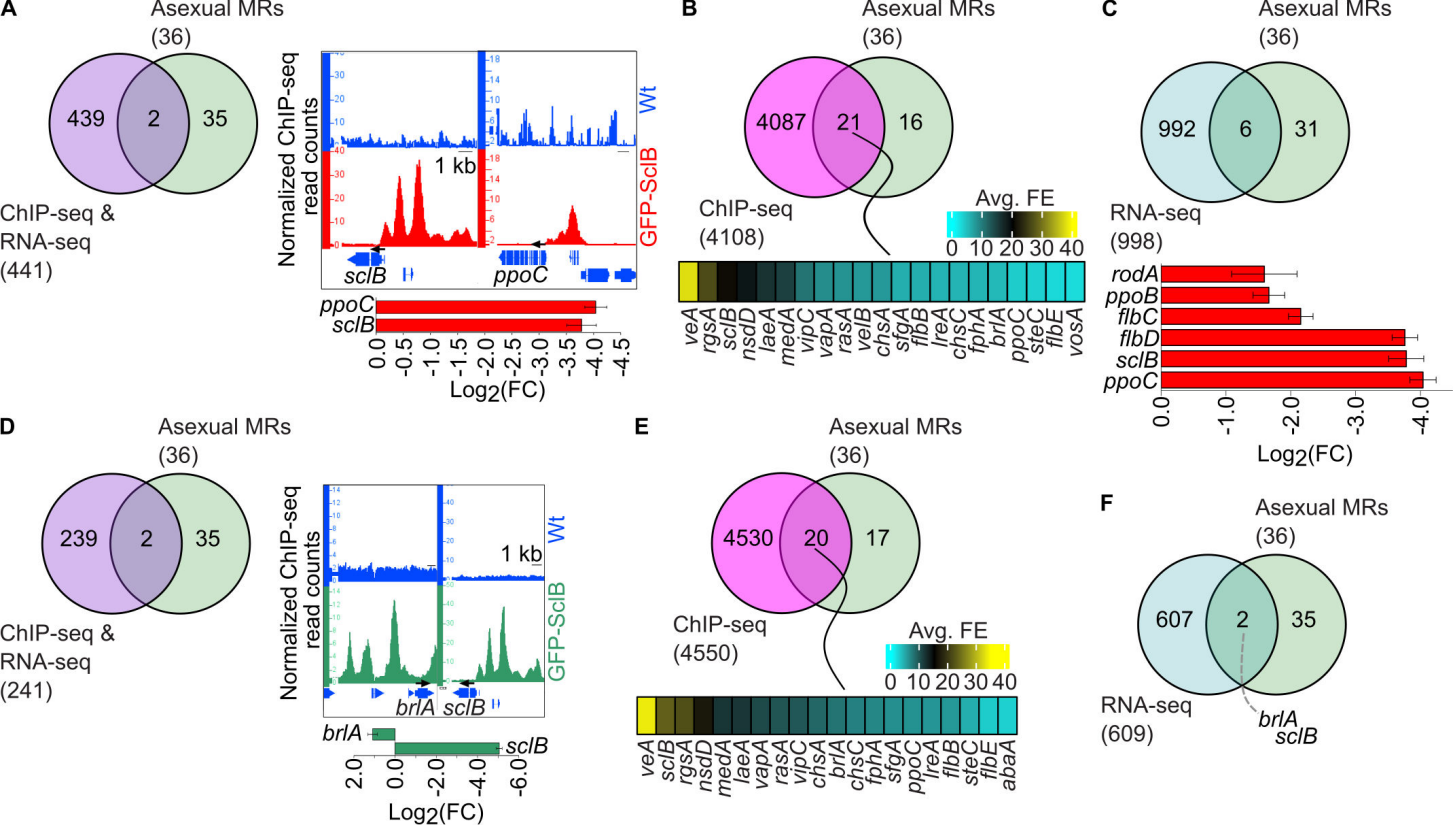
*A. nidulans* SclB exerts direct transcriptional control on its own expression together with the expression of the regulatory gene *brlA* and the *ppoC* gene encoding an enzyme for oxylipin formation. Venn diagrams illustrate the overlap of the direct target genes, as found from ChIP-seq (cutoffs: *P* < 0.05 and F.E. ≥ 2.0) and RNA-seq (cutoffs: *P* < 0.05 and −1 ≥ log_2_FC ≥ 1) experiments, from mycelia grown under (**A**) Veg or (**D**) Asex conditions, with the list of 36 asexual MRs genes. The right parts in panels **A** and **D** show screenshots from the Integrative Genome Browser (hereafter IGB) where peaks (*in vivo* binding positions) of GFP-SclB to the promoters of genes from the overlaps are shown. The bar plots below the IGB screenshots depict the expression of genes as found in the RNA-seq, performed with RNAs derived from mycelia of Wt and the Δ*sclB* strains grown either under Veg or Asex conditions. Venn diagrams presenting the overlap between the strong ChIP-seq targets, performed with mycelia derived from either (**B**) Veg or (**E**) Asex growth, with the list of asexual MRs genes. The heatmaps under the Venn diagrams depict the strength (as F.E. color key) for the *in vivo* binding of GFP-SclB to the promoters of the 21 (in panel **B**) and the 20 (in panel **E**) genes found in the corresponding overlaps. Venn diagrams show overlaps between the strongly differentially expressed genes, as derived from RNA-seq, with RNAs from mycelia from Wt and Δ*sclB* strains grown under (**C**) Veg or (**F**) Asex conditions, with the list of the asexual MRs genes. The bar plot below the Venn in panel **C** illustrates the expression (as of log_2_FC) of the six genes found in the corresponding overlap.

Different overlaps with the list of the asexual MRs and the Veg and Asex NGS data sets of SclB were performed. Independently, from the growth condition, there was always a very small set of genes, *sclB*, *ppoC,* and *brlA,* from known asexual MRs, that was found to be directly regulated by SclB (Fig. 3A and D). However, the number of genes encoding asexual MRs was larger when the overlaps were performed with genes which only appeared in the ChIP-seq lists (Fig. 3B and E). When overlaps were performed with the lists of DEGs from the RNA-seqs, it was again a small set of genes that emerged as common loci IDs (*brlA*, *sclB, ppoC*, *sclB*, *flbD*, *flbC*, *ppoB,* and *rodA*). In summary, SclB shows a rather strict selective behavior during the transition of mycelia from vegetative to asexual growth, concerning the transcriptional control of genes encoding key regulators of asexual development. Genes coding for the prominent master regulators, *brlA*, *ppoC,* and *sclB* itself, are not only targeted by SclB *in vivo* but also become differentially expressed by it.

### Members of the emericellamides gene cluster are direct targets of SclB during vegetative and asexual growth

**Fig 4:**
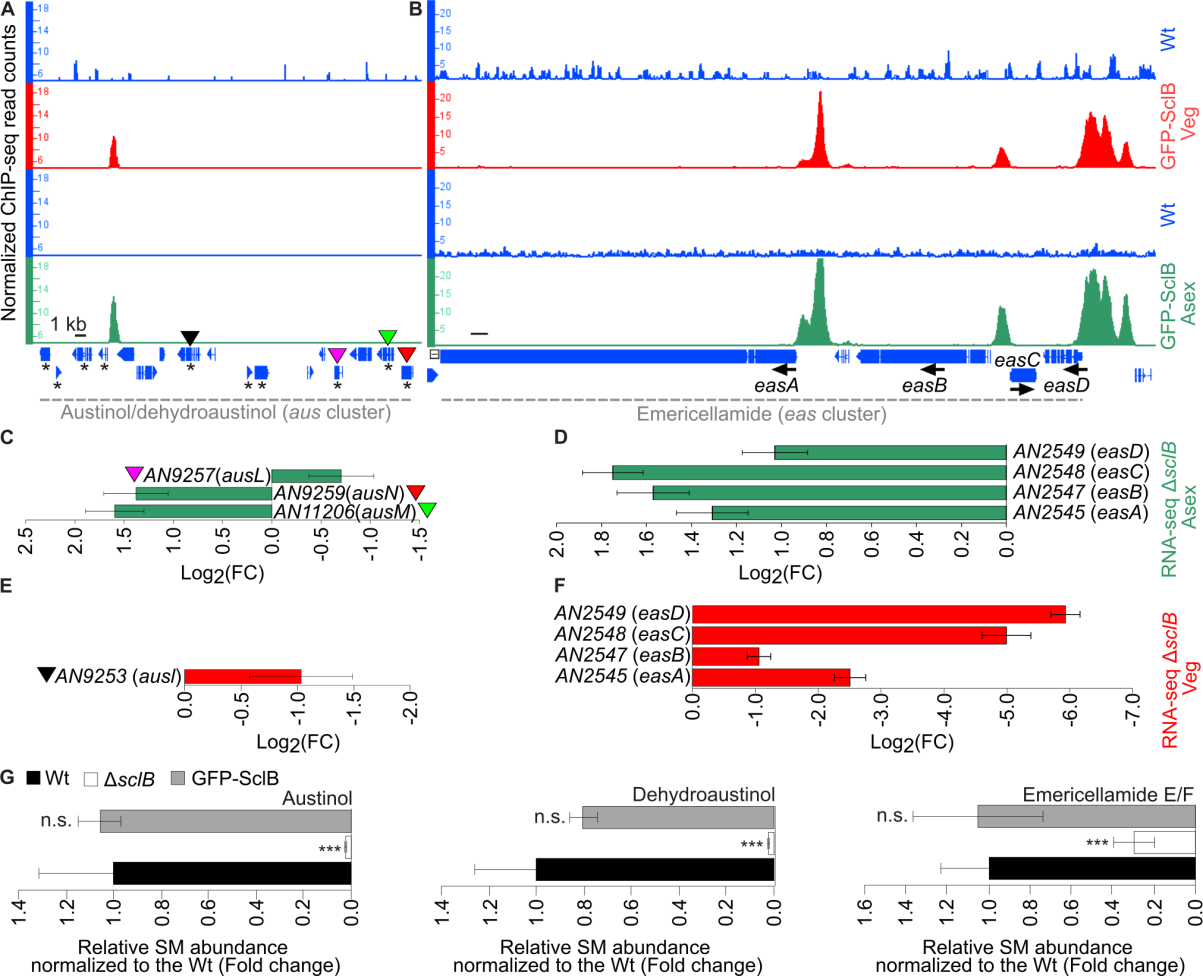
SclB regulates the expression of *A. nidulans* genes of the *aus* and *eas* secondary metabolite gene clusters. Snapshots from the IGB depicting the *in vivo* binding of SclB (ChIP-seq peaks) to promoters of genes of the *aus* (**A**) and *eas* (**B**) gene clusters. Peaks appearing in the red tracks illustrate the *in vivo* binding of SclB from mycelia grown under Veg conditions. Peaks presented in green tracks were derived from mycelia grown in Asex conditions. Blue tracks show the corresponding negative control (background signal) of the ChIP-seq along the corresponding gene clusters in each case. Green bar charts illustrate the expression (as log_2_FC) of genes of the *aus* (**C**) and the *eas* (**D**) clusters, which were found to be differentially expressed (*P* < 0.05) in the RNA-seq data generated with mycelia of the Δ*sclB* and Wt strains, grown in Asex growth conditions. In panels **C** and **E**, the colored triangles show the correspondence of discovered DEGs in the RNA-seq with their specific locus in the ChIP-seq data of panel **A**. The red bar charts show the expression (as log_2_FC) of genes of the *aus* (**E**) and the *eas* (**F**) clusters, found to be differentially expressed (*P* < 0.05) in the RNA-seq data generated with mycelia of the Δ*sclB* and Wt strains, grown under Veg conditions. Bar plots (**G**) illustrate the relative abundance of secondary metabolites, austinol, dehydroaustinol, and emericellamides E/F, normalized to Wt. Each strain is represented as the average and standard deviation of five independent replicates. The detection of secondary metabolites was performed by LC-MS/MS with a charged aerosol detector from extracts derived from the Wt, the *sclB* deletion (Δ*sclB*), and the GFP-SclB strain grown asexually for 3 days on minimal medium plates at 37°C. All three metabolites were detected as m/z in the positive mode. Statistics performed by Student’s t test: ***P ≤ 0.01 and n.s.: not significant.

The zinc cluster transcriptional regulator SclB has been strongly associated with the regulation of secondary metabolism in several Aspergilli. *A. nidulans* SclB is inducing the biosynthesis of emericellamides (23) during vegetative growth and austinol/dehydroaustinol under asexual growth conditions (18). Previous work shows that the corresponding *scl2* ortholog of *A. niger* is controlling the biosynthesis of indoloterpenes/aurasperones (20). It was further investigated whether there is a direct transcriptional SclB control in *A. nidulans* toward members of the *eas* and *aus* biosynthetic gene clusters (BGCs) during the transition from vegetative growth to asexual development. These BGCs are responsible for the biosynthesis of emericellamides, austinol, and dehydroaustinol, respectively. The ChIP-seq data from both experimental conditions were searched for the presence of peaks in promoter regions of genes of the *eas* and *aus* clusters. In the case of the *aus* BGC, one clear peak was discovered under both experimental conditions (Fig. 4A). However, examination of the corresponding transcriptomic data showed that the *aus* genes, which were differentially expressed, were not in close proximity to the SclB location *in vivo* (Fig. 4C, E, and A). Moreover, strong ChIP-seq peaks were identified under both conditions at the promoters of all four genes (*easA-D*) coding for members of the *eas* BGC for emericellamides production (Fig. 4B). The expression of the *eas* genes during Asex growth was found to be repressed by SclB (Fig. 4D). In contrast, the expression of the same genes was found to be induced by SclB during vegetative growth (Fig. 4F). This corroborates that SclB is directly associated with promoters of genes coding for BGCs *in vivo*. Furthermore, the regulatory role of SclB changes during the transition from vegetative growth to asexual development, from being inductive to becoming repressive concerning the *eas* genes in particular. Lastly, to explore whether the discovered direct *in vivo* regulation that SclB exerts on *aus* and *eas* BCGs is of biological relevance for the fungus, the levels of the corresponding metabolites produced by these clusters were assessed. Extracts of secondary metabolites from Wt, Δ*sclB,* and GFP-SclB strains, from colonies grown on solid medium for 3 days under asexual growth conditions, were used for the profiling of these metabolites. The synthesis of austinol and dehydroaustinol produced by the *aus* BCG, as well as the emericellamides E/F produced by the *eas* BCG, was found to be severely suppressed in the Δ*sclB* strain compared to Wt (Fig. 4G; Table S4). In all cases, the levels of the corresponding secondary metabolites in the GFP-SclB strain (used in the ChIP-seqs) had similar levels as in the Wt. In conclusion, these results clearly illustrate that SclB directly influences austinol/dehydroaustinol and emericellamide synthesis during asexual development.

### *veA* and *velB* are direct target genes of SclB

**Fig 5:**
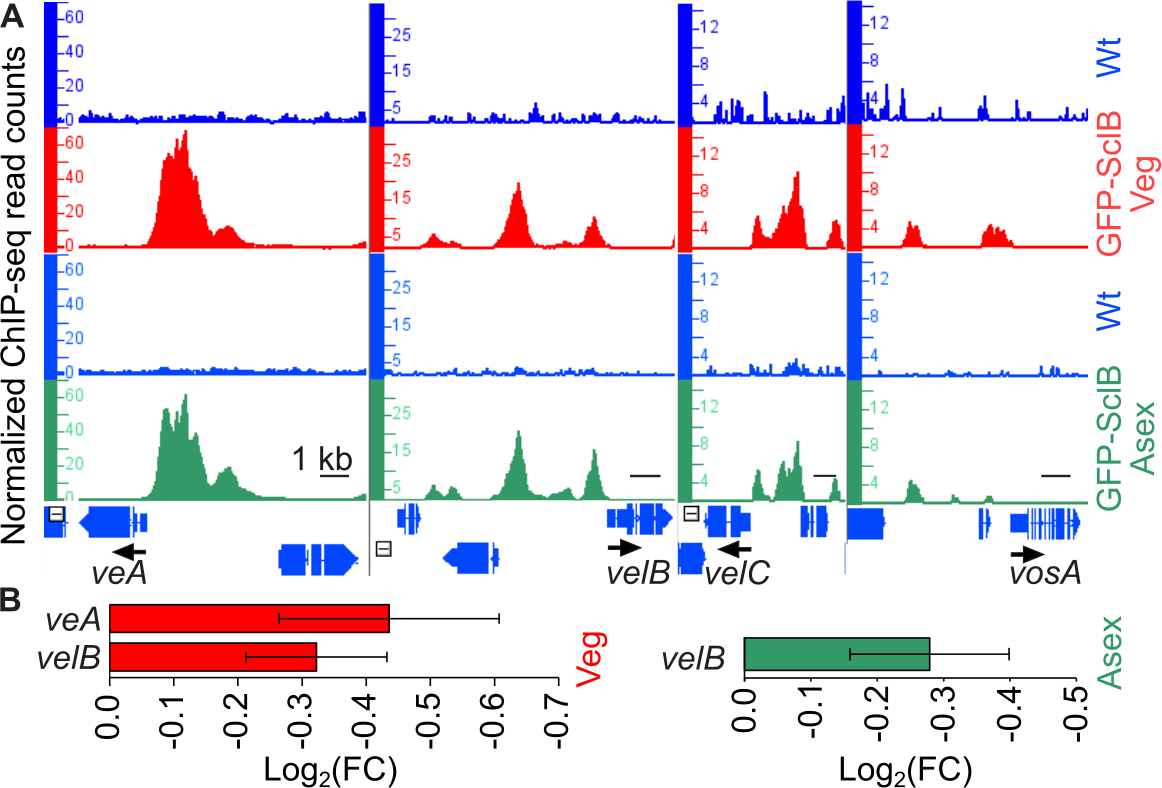
Expression of fungal *veA* and *velB* regulatory genes is induced by the *in vivo* association of SclB to their promoters. (**A**) Screen shots from the IGB depicting the *in vivo* binding of SclB (ChIP-seq peaks) to the promoters of the velvet genes (*veA*, *velB*, *velC,* and *vosA*). Peaks appearing in the red tracks illustrate the *in vivo* binding of SclB in mycelia grown under Veg conditions. Peaks shown in green tracks illustrate *in vivo* binding of SclB in Asex mycelia. Blue tracks show the corresponding negative control (background signal) of the ChIP-seq in each case. (**B**) Bar charts, below the ChIP-seq snapshots, illustrate the expression of *veA* and *velB*, as found to be differentially expressed in the RNA-seq data (*P* < 0.05) generated from mycelia of the Δ*sclB* and Wt strains, grown either under Veg (red bars) or under Asex growth conditions (green bars).

The *A. nidulans* velvet-domain proteins and their orthologs in other species have been associated with a great variety of aspects of fungal development (1). Each of the four velvet family members (VeA, VelB, VelC, and VosA) has been linked to distinct processes in the life cycle of the fungus. VosA was found to be directly associated with *sclB in vivo* (11). A later study linked VosA binding to the transcriptional regulation of the *sclB* gene (18). It appeared that VosA is a repressor of *sclB*, at least in asexually grown fungal colonies. We hypothesized that the regulation between VosA and SclB can be (i) mutual and (ii) possibly SclB can transcriptionally control other velvet genes besides *vosA*. To test this, SclB ChIP-seq data were searched for peaks near the promoters of the velvet genes. The promoter regions of *veA*, *velB,* and *velC* were found to be occupied by SclB at multiple positions (Fig. 5A). Binding signals (peaks), indicating an *in vivo* association of SclB, were found at several positions in the *vosA* promoter. However, these binding events would rather be characterized as weak and transient, especially compared to the ones found in the promoters of the other velvet genes. Notably, the strong *in vivo* association of SclB to the promoters of *veA*, *velB,* and *velC* was taking place independently from the growth conditions (Fig. 5A). Subsequently, it was examined whether these binding events could provoke changes in velvet gene expression. Investigation of the corresponding RNA-seq transcriptomic data revealed that only two out of four velvet genes, *veA* and *velB*, were repressed in Δ*sclB* compared to the Wt, when fungal mycelia were grown under vegetative conditions (Fig. 5B). This highlights the inductive role of SclB when associated with the promoter regions of the corresponding velvet genes (Fig. 5A). During early asexual growth, only *velB* was repressed in Δ*sclB* compared to Wt (Fig. 5B). These data suggest that SclB binds to the promoters of the *veA* and *velB* genes and presumably supports their activation, whereas the function of the interaction of SclB to the other promoters of velvet genes remains elusive yet.

### The *scl2* ortholog of *sclB* from the plant pathogen *Verticillium dahliae* encodes a protein with partial conserved function with *A. nidulans* SclB

*A. nidulans* uses asexual spores to disseminate and colonize new habitats. In contrast, the plant pathogenic fungus *V. dahliae* produces spores when it reaches the host xylem sap to colonize the upper parts of the plant (24, 25). We wanted to investigate whether SclB is conserved in another filamentous fungus that uses the production of spores in a very distinct environment. BlastP (26) was employed using the amino acid sequence from *A. nidulans* SclB as a reference. We discovered two neighboring genes, VDAG_JR2_Chr8_g03870a and g03890a. None of the deduced amino acid sequences had any predicted domains. It was then assumed that this might be a misannotation; hence, a proposed cDNA transcript of one gene was amplified and sequenced (Fig. S7). The deduced amino acid sequence of *Vd* Scl2 shares approximately 48% identity with *An* SclB (using MUSCLE alignment), and a Zn(2)-C6 fungal-type DNA-binding domain was predicted (IPR001138). The open reading frame of *Vd scl2* was deleted, and the phenotype of the resulting strain was investigated. There were no significant differences in colony morphology or production of conidiospores compared to Wt (Fig. S5).

**Fig 6:**
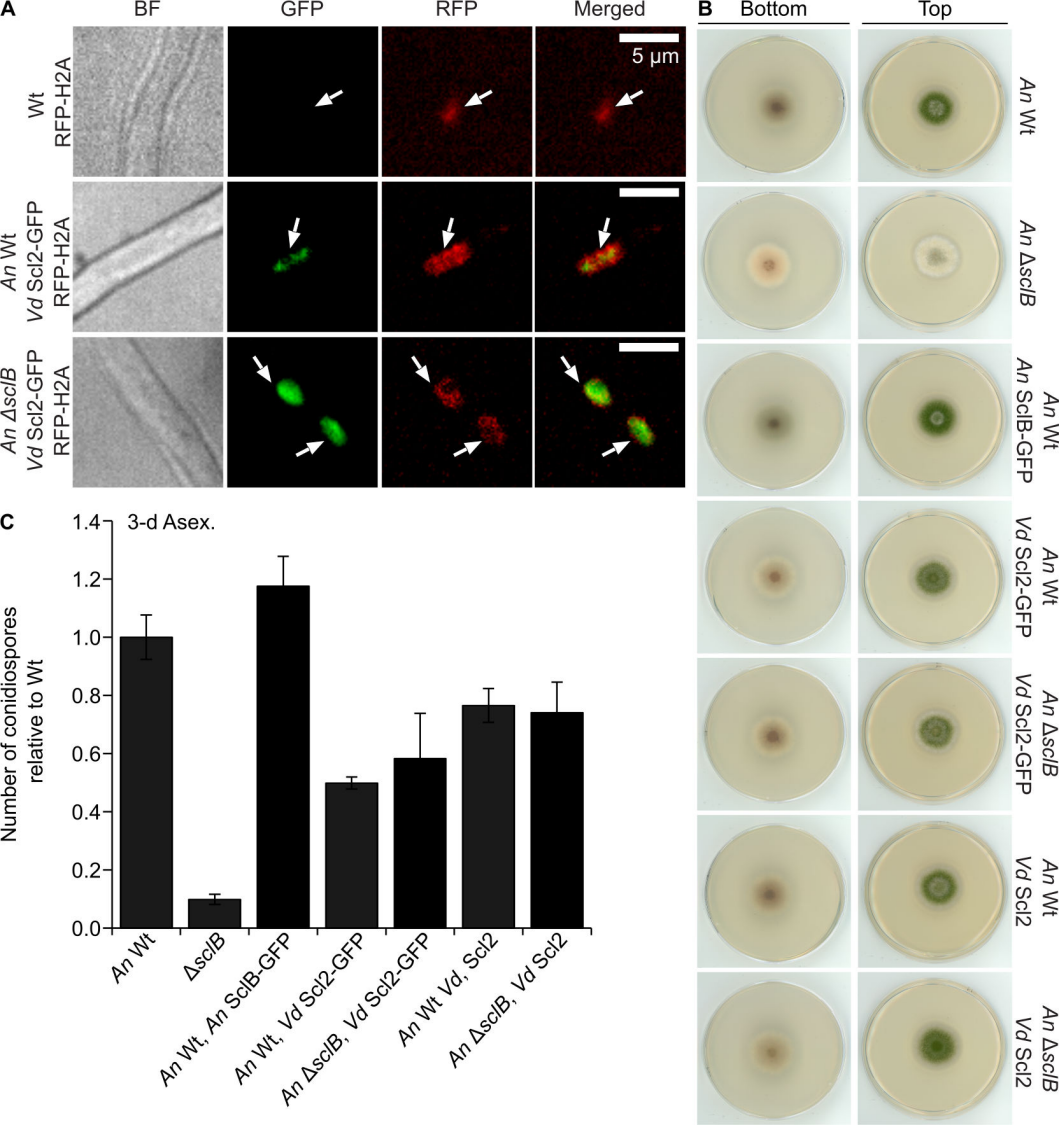
The nuclear Scl2 protein from plant pathogenic *V. dahliae* can overtake SclB regulatory functions and partially rescue the defective phenotype of the *A. nidulans* Δ*sclB* mutant strain. (**A**) Cellular localization of the *Vd* Scl2 tagged with a GFP from *V. dahliae* (*Vd* Scl2-GFP), expressed in the Wt and in the Δ*sclB* strains of *A. nidulans.* In both cases, Scl2-GFP was expressed under the native promoter of *An sclB*. The *An* Wt, the *An* Wt *An* SclB-GFP, and the *An* Δ*sclB An* SclB-GFP strains additionally carried a transgene expressing the nuclear marker H2A tagged with RFP (red fluorescent protein). For confocal images, hyphae were grown under Veg growth conditions. (**B**) Phenotypical characterization of *A. nidulans* strains, grown under asexual growth conditions for 3 days. Each plate was initially inoculated in the middle with a total of 2,000 spores. After a period of 3 days, scans were made from the upper and bottom part of the plates. (**C**) Quantification of the number of asexual spores (conidia), for all the strains presented in panel **B**, after 3 days of growth under asexual conditions (constant light and 37°C). The final quantification results for each strain were derived from a minimum of at least three replicates (plates). Every plate was initially inoculated with a total of 30,000 spores, which were spread all over the plate by using glass beads. Results for the quantification are normalized relative to the Wt strain and represent averages and standard deviations of at least three biological replicates.

It was examined whether the putative *Vd* Scl2 protein might have retained some functions, thus maybe being able to complement the phenotype of the *An* Δ*sclB* strain. Therefore, *A. nidulans* strains were generated in locus expressing *Vd scl2* with or without a C-terminal GFP (green fluorescent protein), in Wt and Δ*sclB* background strains. The nuclear localization of *Vd* Scl2 was subsequently tested by employing confocal live imaging microscopy in the Wt (as a negative control/background signal) and all the complementation strains in which *Vd* Scl2 was tagged with GFP at the C′-terminus. All strains were transformed with the universally used RFP-H2A red nuclear fluorescent marker as a reference for nuclear signal. *Vd* Scl2-GFP, either expressed in the Wt or in the Δ*sclB* strain, was always observed in the nucleus (Fig. 6A).

Next, it was tested whether the nuclear localized *Vd* Scl2 can rescue the phenotype of *An* Δ*sclB*. After 3 days of asexual growth, all the strains carrying *Vd scl2* formed colonies very similar to Wt (Fig. 6B). Quantification of asexual spores revealed that all versions were functional. The amount of the produced conidiospores did not reach Wt levels; however, more spores were produced than in the Δ*sclB* strain (Fig. 6C). In conclusion, these data strongly support that *V. dahliae* Scl2 and SclB from *A. nidulans* are both nuclear localized proteins, with Scl2 in parts functionally complementing the deletion of *sclB* in *A. nidulans*, regarding asexual growth.

## DISCUSSION

Airborne spores produced by fungi are necessary for their propagation and survival in a variety of environments. In filamentous fungi, such as *A. nidulans*, conidiospores are the final products of the asexual developmental program. During that process, several genes coding for essential proteins need to be regulated and coordinated in a temporal and spatial manner. The *A. nidulans* SclB TF is playing a pivotal role in the regulation of gene expression, which is conserved in other filamentous fungi and influences many aspects of asexual development and secondary metabolism (18). However, it is not clear yet how SclB applies its regulatory roles, particularly during the outset of the asexual development. Here, we studied the influence of SclB at the transition of the fungus from the undifferentiated vegetative growth into the early asexual development on a genome-wide scale.

The high-throughput genome-binding experiments for the first time showed that SclB can be associated *in vivo*, via a novel nine-base-pair DNA motif (PSRE), to around ca. 4,000–4,550 genes, which are approximately half of the total genes in the *A. nidulans* genome*,* regardless of the growth condition (Fig. 1; Fig. S1 and Table S3). Moreover, there is a very high correlation among these two data sets, indicating a conserved preference that SclB shows for promoters of specific genes (Fig. 2A). However, these binding events could lead to actual changes in the expression of only a smaller number of genes, 441 for Veg and 241 for Asex growth correspondingly (Fig. 1; Fig. S1 and Table S3). This could imply further roles that SclB might have during the particular shifting, related to responses to other external or internal inputs that might accompany this transition. Future studies are necessary to further elucidate yet uncovered developmental roles of SclB in fungal life.

A major function of SclB in the developmental transition is the control of the key regulatory genes *ppoC*, *brlA*, *veA,* and *velB,* combined with an auto-control of *sclB* itself (Fig. 3 and 5). The direct transcriptional autoregulation of SclB to itself and the control of the gene for the dioxygenase PpoC are two novel discoveries of the complex SclB regulatory network that were hidden so far. Specifically, the implication of SclB in the synthesis of the oleic acid-derived psi factor psiBβ, a known inducer of asexual growth (1, 16), highlights the variety of control levels via which SclB can influence the initiation of asexual growth. It has been shown that *brlA* expression is induced when SclB is overexpressed in mycelia growing for 24 h vegetatively (18). Moreover, it is also known that *brlA* induction starts at least 5 h post-induction of asexual development (10). SclB keeps the expression of *brlA* at a low level at least up to the point of 3 h post-induction. Our study supports that SclB maintains first a low *brlA* expression until the first 3 h of asexual growth induction (Fig. 3). This illustrates the finding of a subtle fine-tuning that SclB asserts toward BrlA as part of the developmental transition. In the future, it will be informative to examine what the actual effect of SclB on the expression of *brlA* is in later time points, such as 5 h and more post-induction. This study has established a novel transcriptional relationship between SclB and the velvet domain regulatory genes *veA* and *velB*, coding for regulators with opposite roles in asexual growth (13–15). In fact, SclB is able to induce the expression of both velvets under vegetative conditions, but after the shift to asexual growth, its inductive action was delivered only to *velB* (Fig. 5). This is in line with the promoting role of VelB during the asexual growth of the fungus. The MsnA regulator of development binds to the *sclB* promoter *in vivo* and induces gene expression for vegetative and asexual growth (27). SclB is strongly associated with multiple *msnA* promoter sites during Veg or Asex growth conditions (Fig. S6). However, none of these binding events led to differential expression of *msnA* (Table S3). It is yet elusive whether these direct promoter interactions might result in transcriptional control of the *msnA* promoter at different time points than the one tested by our RNA-seqs.

Chemical compounds derived by fungal secondary metabolism can trigger developmental processes but also shape the interactions of the fungus with other living organisms nearby (28). SclB affects *in vivo* the expression of genes belonging to *eas* and *aus* gene clusters (Fig. 4), responsible for the synthesis of emericellamides (compounds with antibiotic action) (23) and austinol/dehydroaustinol (important in sporulation) (29, 30) correspondingly. This direct transcriptional influence of SclB is also directly affecting the synthesis of the corresponding secondary metabolites during asexual growth (Fig. 4). This type of regulation shows that changes in secondary metabolites triggered by SclB are necessary during the passage of the fungus from vegetative to asexual growth.

A previous study has shown that *A. fumigatus sclB* (*Afu6g11110*) is sharing 55% amino acid sequence similarity with *A. nidulans sclB,* and the corresponding deletion strain showed Wt-like development. However, the severe phenotype of the Δ*sclB* strain in *A. nidulans* was fully complemented by the *sclB* ORF from *A. fumigatus* (18). In our study, we further examined whether the function of *A. nidulans* SclB could be functionally complemented by homologs from other ascomycete species with different lifestyles and colonization environments, compared to *A. nidulans* or *A. fumigatus*. Although the deletion of *scl2* from the plant fungal pathogen *V. dahliae* did not show any developmental defects (Fig. S5), the heterologous expression of *V. dahliae* Scl2 in *A. nidulans* Δ*sclB* showed a partial rescue of the severe phenotype of the deletion strain, highlighting a partial functional compatibility among the homologs (Fig. 6). SclB/2 in the soil inhabitant *A. nidulans* as well as in the vascular pathogen *V. dahliae*, which is overwintering *ex planta* in the soil, seems to operate, at least partially, in a similar manner. It will be interesting to examine in the future in more detail to what degree the targets of *Vd* Scl2 and the *An* SclB are alike.

**Fig 7:**
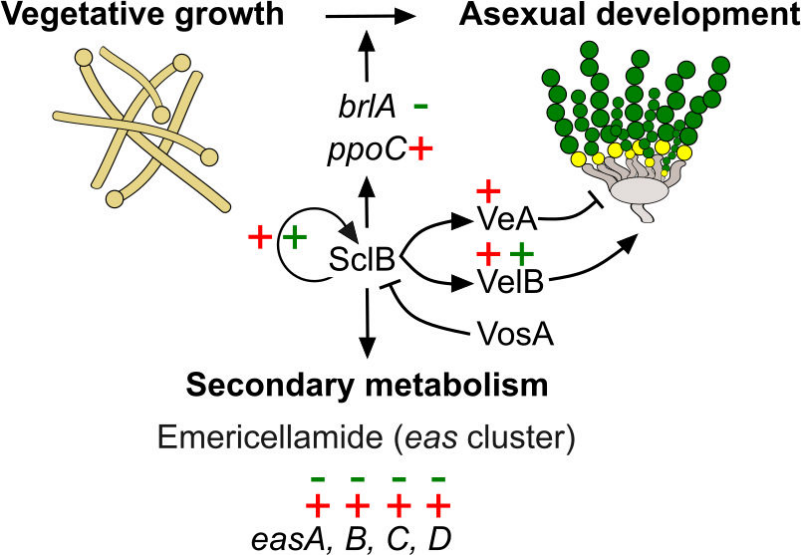
SclB is the fungal organizer and coordinator for the transition from vegetative to asexual growth. The graphic illustrates a current model where SclB orchestrates the shift toward asexual reproduction. BrlA, PpoC, and SclB are specific established master regulators of asexual development that are transcriptionally controlled by SclB during this transition directly. Additionally, SclB directly influences the transcription of *veA* and *velB* at the vegetative phase. However, the expression of *velB* (encoding the VelB asexual inducer) but not of *veA* (encoding the VeA asexual suppressor) continues to be induced shortly after the transition to the asexual phase. SclB is also associated with all four genes (*easA, B, C,* and *D*) of the *eas* cluster *in vivo*, impacting directly the synthesis of emericellamides. The plus or minus signs beside the genes indicate either induction or repression of gene expression correspondingly, mediated by SclB. Signs of + or – in red refer to Veg and those in green refer to Asex conditions, as derived from the combined ChIP-seq and RNA-seq data. The designing of the graphical model was performed with the use of the freely available vector graphics software Inkscape (https://inkscape.org/).

In summary, our data are presented in a model (Fig. 7), which places SclB in a prominent position in a signaling pathway, where it orchestrates and promotes the passage of the fungus from undifferentiated vegetative growth to asexual development. SclB ensures the activation of several mandatory regulatory circuits necessary for the onset of conidiation by its direct *in vivo* association with promoters of key regulatory genes, such as *brlA*, *ppoC,* and *sclB*. Our work further establishes SclB as a main actor in the CDP for asexual sporulation. Asexual spore formation is an essential step for ascomycetes to spread, and a better knowledge of the control of the fungal SclB regulatory function might contribute to better control of fungal growth and dispersal in different environments.

## MATERIALS AND METHODS

### Strains, media, and growth conditions

All *E. coli*, *A. nidulans,* and *V. dahliae* strains used in this study are listed in Table S1. As Wt strains *A. nidulans* AGB551 (31) and *V. dahliae* JR2 were used (32).

Minimal medium for *A. nidulans* had the composition: 1% (wt/vol) glucose, 2 mM MgSO_4_, 1× AspA (7 mM KCl, 70 mM NaNO_3_, 11.2 mM KH_2_PO_4_, pH 5.5), 0.1% (vol/vol) trace element solution (76 µM ZnSO_4_, 178 µM H_3_BO_4_, 25 µM MnCl_2_, 18 µM FeSO_4_, 7.1 µM CoCl_2_, 6.4 µM CuSO_4_, 6.2 µM Na_2_MoO_4_, and 174 µM EDTA), and pH 5.5 (33). For a solid medium, 0.1% (wt/vol) uracil and 2% agar were added. Liquid medium was supplemented with 5 mM uridine and 0.1% (vol/vol) pyridoxine. *A. nidulans* protoplasts were grown on solid medium supplemented with 120 mg/mL nourseothricin for selection. Selection-marker cassettes were recycled while growing the strains on medium with 0.5% (wt/vol) xylose/0.5% (wt/vol) glucose. Strains were grown at 37°C.

*V. dahliae* strains were cultivated and transformed as described previously by Harting et al. (34).

Cloning was performed with *E. coli* DH5α. A lysogeny broth (LB) (35) was the liquid culture medium (1% tryptone, 0.5% yeast extract, 1% NaCl, supplemented with 100 µg/mL ampicillin for selection) used for the propagation of *E. coli* cells transformed with plasmids according to reference (36). For a solid medium, 2% (wt/vol) agar was added. *E. coli* strains were grown at 37°C.

### Plasmid manipulations and cloning

The backbone plasmid for construction of *A. nidulans* transformation cassettes was pME4696, initially used by Meister et al. (37). This vector carries a *Pml*I and a *Swa*I restriction site for integration of the 5′- and 3′-flanking regions of the target gene. These flanking parts dictate the exact position in the *A. nidulans* genome where the cassette is integrated. Amplification of 5′- and 3′-regions was performed using genomic DNA (gDNA) from the AGB551 strain. Assembly of the amplicons with pME4696 was performed by employing the GeneArt Seamless Cloning and Assembly Kit (Invitrogen, Carlsbad, CA, USA). All final cassettes carried nourseothricin resistance cassettes (natRM), as a recyclable marker. Isolation of plasmid DNA was conducted by the NucleoSpin Plasmid Kit (Macherey-Nagel, Düren, Germany) according to the manufacturer’s instructions. Prior to excision of the cassette, all plasmids were verified via Sanger sequencing by Microsynth Seqlab GmbH (Göttingen, Germany).

### Assembly of the *Vd scl2::hinge::GFP::3′UTR_AnsclB_::trpC-*terminator cassette and the *Vd*-Scl2-GFP *A. nidulans* strain

The *Vd scl2* was initially amplified from gDNA template by using the primers RH835/836. The PCR product was subsequently cloned into pJet (pME5569) and sent for sequencing. The exact annotated sequence of the gene is presented in Fig. S7**.** It followed the junction of the *Vd scl2* with the linker, GFP, and the marker. Fragment-1 consisted of a 5′-flanking region (amplified from gDNA template), and the gene (derived from the previous pJet vector) was constructed by using RH838/844. Fragment-2, composed of the linker, GFP, and the marker, was amplified from the plasmid pME5072 (38) with the primers AO165/ML8. The third fragment was generated by a 3′-flanking region, from gDNA template amplified with the primers RH840/841. All three fragments were subsequently cloned via Seamless reaction into the backbone vector pME4548 (39), previously digested by *Stu*I and *Eco*RV. The final plasmid was named pME5567.

It followed the cloning of the cassette for the strain *Vd* Scl2-GFP of *A. nidulans*. The 5′-flanking part was composed of four fragments: a 1,898 bp amplicon spanning from the promoter of *An sclB* till the end of the gene’s *5′-UTR* (MB1569/1570), a 3,234 bp amplicon for the *Vd scl2::hinge::GFP* (MB1571/1572), a 637 bp fragment starting from the start of the *3′-UTR* of the *An sclB* (MB1573/1574), and a 716 bp amplicon for a *trpC* terminator (MB1575/1576). The templates for each amplification were as follows: for the first and the third part AGB551(Wt) gDNA, for the second was the plasmid pME5567 carrying the coding sequence of *Vd scl2* from *V. dahliae* fused with a linker (hinge) and a GFP, and for the fourth amplicon the plasmid pChS242 (40) was used carrying the sequence of the *trpC* terminator. The junctions of all parts constituting the whole 5′-flanking part took place by fusion PCR. The 5′-flanking part of *sclB* was integrated via *Pml*I into pME4696. The 1,510 bp 3′-flanking region was amplified from gDNA of AGB551 with the primers (MB1577/1578), which was then introduced into the *Swa*I site of the same vector that carries the 5′-flanking part. The final plasmid after the incorporation of both flanking regions was named pME5564. Finally, the cassette was excised with the *Mss*I (*Pme*I) restriction enzyme and used subsequently for transformation in AGB551(Wt) and AGB1007(Δ*sclB*) *A. nidulans* strains, leading to the corresponding strains AGB1711 and AGB1713, after the recycling of the selection marker.

### Assembly of the *Vd scl2::3′UTR _AnsclB_:trpC-*terminator cassette and the *Vd*-Scl2 *A. nidulans* strain

The construction of this cassette was performed using the same sets of primers and templates as used for the previous cassette *Vd scl2::hinge::GFP::3′UTR_AnsclB_::trpC-*terminator. The only difference was the set of primers used to amplify the *Vd Scl2* from the corresponding vector that carried the coding sequence of *Vd scl2* fused with the *linker* and the *GFP* sequence. In that case, the set of primers MB1571/1579 was used (instead of MB1571/1572), where specifically the reverse primer (MB1579) ends the corresponding amplicon just after the stop codon of the *Vd scl2,* and it is also designed to be fused directly with the *3′UTR _AnsclB_* with a fusion PCR. Once the plasmid has incorporated its 5′- and 3′-flanking regions, named pME5565. The cassette was then excised and transformed into AGB551(Wt) and AGB1007(Δ*sclB*) *A. nidulans* strains, which, after the recycling of the selection marker, led to the AGB1715 and AGB1717 strains, respectively.

### Assembly of the Δ*Vd scl2* cassette and the Δ*Vd scl2 V. dahliae* strain

The 5′ (1,211 bp, primers RH838/RH839) and 3′-flanking regions (1,000 bp, primers RH840/RH841) were amplified from Wt gDNA. The Nourseothricin resistance marker cassette (2,194 bp, primers ML8/ML9) was amplified from pME4815 (41). The fragments were fused to pME4548, which was restricted with *Stu*I and *Eco*RV before using the GeneArt Seamless Cloning and Assembly Kit. The resulting plasmid pME5566 was transformed into the *V. dahliae* Wt(JR2) strain to get the VGB0685 strain.

### Phenotypical assays

For examination of *A. nidulans* colonies, 2,000 spores of each strain were spotted on MM plates. After 3 days of asexual growth, plates were scanned from top and bottom. *V. dahliae* phenotyping was performed by spotting 5 × 10^4^ spores of each strain on pectin-rich SXM; colonies were pictured after 5 days.

Quantification of *A. nidulans* conidia was performed with fresh spores from which identical numbers were distributed equally on MM plates. After 3 days of asexual growth, the spores were collected and quantified. The quantification of *V. dahliae* spores was done as described before Starke et al. (41). The spores of both fungi were counted with the Coulter Z2 particle counter (Beckman Coulter GmbH, Krefeld, Germany).

### DNA and RNA extraction

Genomic DNA was extracted from mycelia grown overnight in light and at 37°C. DNA isolation was performed as mentioned by Thieme et al. (18).

Liquid cultures were inoculated with 10^8^ spores in 100 mL medium. After 20 h of growth with constant agitation in light, vegetative mycelia were dried, and 100 mg for each sample was snap-frozen in liquid nitrogen. For the Asex RNA-seq, mycelia were grown vegetatively for 20 h and then transferred to MM plates for another 3 h. Mycelia were processed as described before. The extraction of RNAs was done as described in Bastakis et al. (27).

### Southern hybridization

All constructed strains were checked for correctness by Southern hybridization (42). The labeling of the Southern’s probes was done by AlkPhos Direct Labeling Module (GE Healthcare Life Technologies, Little Chalfont, UK) following the manufacturer’s instructions.

### ChIP-seq

The GFP-SclB (AGB1010) strain of *A. nidulans*, expressing *sclB* fused in its N′-terminus to the GFP under its native promoter, was used to perform ChIP-seq experiments under Veg and Asex conditions. A total number of 5 × 10^8^ spores was inoculated in 500 mL liquid medium using 2 L flasks and grown for 20 h under constant rotation in the presence of light at 37°C. For the Veg ChIP, after the 20 h incubation, mycelia were dried and immersed in 1% formaldehyde fixing solution for 20 min. For the 2nd ChIP, mycelia were transferred after the 20 h vegetative growth to MM plates and grown asexually for an additional 3 h. Fixation was performed as previously described. For the Veg ChIP, three biological replicates were used for GFP-SclB and the Wt strain (negative control/background signal) correspondingly. For the Asex ChIP, three biological replicates for GFP-SclB and two for the Wt strain were used. In the subsequent analysis for the identification of the peaks, the sequenced data for the second biological replicate of the Wt were used as a control for the second and the third biological replicates of the GFP-SclB sequencing data. The GFP antibody (Abcam ab290) was applied to all IPs. The rest of the ChIP protocol, library preparation, NGS sequencing, and ChIP-seq data analysis were performed as described by Sasse et al. (40). ChIP-seq library constructions and sequencing of the ChIP samples were performed in the NGS-Integrative Genomics Core Unit (NIG), University Medical Center Göttingen.

The pipeline for the ChIP-seq analysis was as described by Sasse et al. (40). Web tools for the analysis were provided either from the GALAXY (43) or the RStudio platforms, both maintained by the GWDG (Gesellschaft für wissenschaftliche Datenverarbeitung mbH Göttingen). In short, the Bowtie2 tool (44) was used for the mapping of the reads derived from the sequencing against the reference genome of *A. nidulans* (FungiDB-46_AnidulansFGSCA4_Genome.fasta). The MACS2 tool (45) was employed for the discovery of statistically significant peaks. MACS2 normalizes the data, before identification of statistically significant peaks, by scaling the control sample (Wt in our case) and the actual test sample (GFP-SclB in our case) to the same sequencing depth, taking into account background modeling via dynamic Poisson statistics. ChIP-seq peaks were visualized in the IGB (46), using bigwig files generated by the bamCoverage tool of the deepTools2 package (47). The distribution of the identified peaks over different genomic elements was assessed using the ChIPseeker R package (48), and for the *de novo* motif discovery of the PSRE motif, the MEME-ChIP tool (49) was employed. The ShinyGo v0.82 tool (50) was used for the GO-enrichment analysis, the InteractiVenn (51) for the Venn diagrams, and the CorrPlot R package for the visualization of the Pearson correlation coefficient (52).

### RNA-seq and data analysis

The preparation of the RNA-seq libraries and the following sequencing, from the step of the quality check of the initial RNA samples till the quality of sequencing, followed the pipeline as described by Szemes et al. (53)**.**

The RNA-seq analysis was performed on the GALAXY platform (43), as provided by GWDG. Raw sequencing reads were mapped in the *A. nidulans* genome (downloaded from fungidb.org: FungiDB-46_AnidulansFGSCA4_Genome.fasta) by using the Bowtie2 (44) (Galaxy Version 2.3.4.2). Subsequently, matrices were prepared by employing the htseq-count tool (54) (Galaxy version 0.9.1), which were then used to calculate the DEGs of the deletion Δ*sclB* versus the Wt samples, with the Galaxy implemented tool DESeq2 (55) (Galaxy version 2.11.40.6+galaxy2).

### Microscopy

Fluorescence microscopy was performed as described by Bastakis et al. (27). The nuclear localization of the *Vd* Scl2-GFP and GFP-SclB was assessed by the expression of RFP-H2A. The integration of the gene encoding the nuclear marker was performed into the corresponding *A. nidulans* strains by the transformation of the cassette *^p^gpdA::intron::mrfp::h2A*(*cDNA*) carried by plasmid pME3173 (56), resulting in the strains, AGB1718, AGB1719, AGB1720, and AGB1721 (Table S2).

### Secondary metabolites profiling

Initially, plates with solid medium were prepared, upon which an equal number of spores was point-inoculated. For each strain, five plates were prepared (five independent replicates). Colonies were left to grow at 37°C for 3 days under asexual growth conditions. Extraction of secondary metabolites and the subsequent LC-MS/MS analysis were performed as described by Liu et al. (57). The software employed for the corresponding analysis was FreeStyle 1.6 (Thermo Fisher Scientific). Details for the detected secondary metabolites of austinol, dehydroaustinol, and emericellamides E/F are presented in Table S4.

## Data Availability

The raw sequencing data for the ChIP-seqs and RNA-seq have been deposited in the NCBI Sequence Read Archive (SRA) under the BioProject IDs PRJNA1292721 and PRJNA1293339.

## SUPPLEMENTAL MATERIAL

Supplemental figures mbio.03488-25-s0001.pdf Fig. S1 to S7.

Table S1 mbio.03488-25-s0002.xlsx Primers used in this study.

Table S2 mbio.03488-25-s0003.xlsx Strains used in this study.

Table S3 mbio.03488-25-s0004.xlsx Target genes from the ChIP-seq experiments and differentially expressed genes from the RNA-seq experiments.

Table S4 mbio.03488-25-s0005.xlsx Details for the detected secondary metabolites.

ASM does not own the copyrights to Supplemental Material that may be linked to, or accessed through, an article. The authors have granted ASM a non-exclusive, world-wide license to publish the Supplemental Material files. Please contact the corresponding author directly for reuse.
