# Supplementary material for: The Aspergillus nidulans transcription factor SclB governs the transition from vegetative to asexual development
Source: mBio. 2026 Feb 25;17(4):e03488-25. doi: 10.1128/mbio.03488-25 (PMC13059802; doi:10.1128/mbio.03488-25)
Supplement: Supplemental figures — Fig. S1 to S7. [file mbio.03488-25-s0001.pdf]

Supplemental Material for

The *Aspergillus nidulans* transcription factor SclB governs the transition from vegetative to asexual development

Emmanouil Bastakis[1], Rebekka Harting[1], Alexandra Scheel[1], Tanja Lienard[1], Christoph Sasse[2], Merle Aden[1], Gabriele Heinrich[1], Verena Grosse[1], Nicole Scheiter[1] and Gerhard H. Braus[1*]

[1] Department of Molecular Microbiology and Genetics, Institute for Microbiology and Genetics, University of Göttingen, Göttingen, Germany.

[2] Department of Prosthodontics, University Medical Center, Göttingen, Germany.

[*]corresponding author

Email: gbraus@gwdg.de (GHB)

This pdf file includes:

FIG S1 to S7

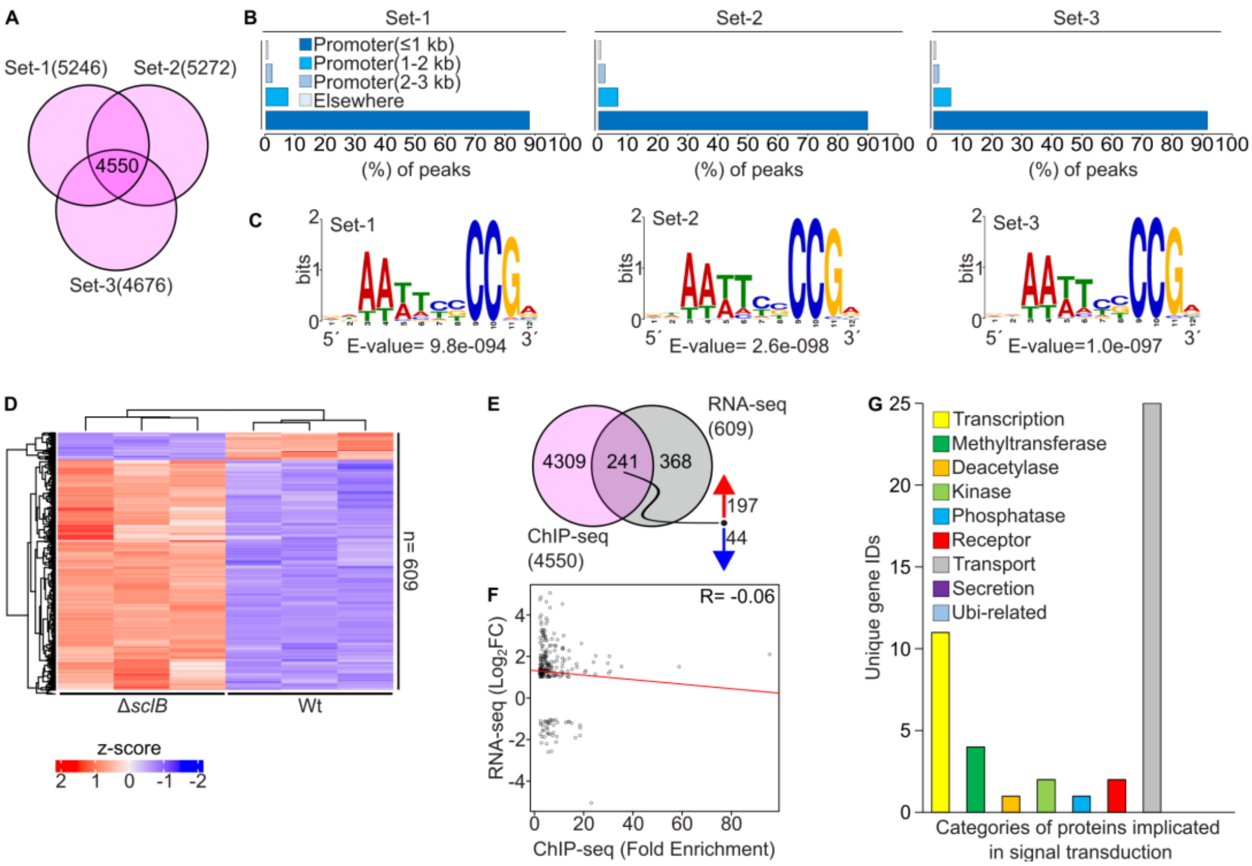

FIG S1: *In vivo* binding landscape of the GFP-SclB transcriptional regulator during early *A. nidulans* asexual development. (**A**) The Venn diagram presents the overlap of three independent sets of ChIP-seq analysis for GFP-SclB versus Wt. ChIP-seq was performed in light under Asex growth conditions. In sum, 4550 unique gene locus IDs, found to have peaks (cut offs: $p < 0.05$ and fold enrichment (F.E.) ≥ 2.0) located in up to 3 kb promoter regions, discovered at the same time in all three independent sets of the ChIP-seq samples. (**B**) Bar diagrams depict the distribution of the statistically significant ChIP-seq peaks over different genetic structures for each of the three independent sets of this analysis. For all three sets around 90 % of the discovered peaks are located within promoter regions of genes up to 1 kb from the transcriptional start site. (**C**) Top scored logos, as discovered by performing a *de novo* motif analysis by employing the MEME-ChIP tool. For each independent set of analysis, as an input a set of 150 sequences was used, each with 100 bp length. All of these sequences were located under the summits of the top-ranked 150 ChIP-seq peaks for each set. The summits of all of these peaks were located up to 3 kb promoter regions. (**D**) The heatmap illustrates differential expression of genes (as z-score values) from mRNAs of Δ*sclB* and Wt strains, derived from Veg grown mycelia. The number n=609 represents the total number of genes that were differentially expressed under the cut offs: $p < 0.05$ and $-1 \geq \log_2$FC(Fold Change) ≥ 1. The RNA-seq was performed with three independent biological replicates of the Δ*sclB* and for the Wt strain. (**E**) Venn diagram presenting the overlap among the highly reproducible targets genes from the ChIP-seq with GFP-SclB (**A**) with the DEG from the RNA-seq of Δ*sclB* (**D**). Both experiments were performed with mycelia from Asex growth conditions. The corresponding cut offs for the ChIP-seq were: $p < 0.05$ and fold

enrichment (F.E.) ≥ 2.0 and for the RNA-seq: $p < 0.05$ and -1 ≥ $\log_2$FC(Fold Change) ≥ 1. The numbers at the right side of the red and blue arrows indicate the total number of genes with an expression that was increased or decreased, from the total genes of the overlap. The set of these 241 gene locus IDs is characterized as the direct *in vivo* targets of GFP-SclB during early asexual development. (**F**) Pearson correlation coefficient, presented by a scatter plot, among the peak`s fold enrichment from the ChIP-seq performed in Asex growth conditions with the gene expression (as of $\mathrm{Log_2}$FC) from the RNA-seq, under the same conditions. For this plot only the 241 direct target genes as discovered by the overlap of ChIP-seq with the RNA-seq in (**E**) were used. The R= -0.06 at the upper right corner of the plot depicts the negative correlation among the compared NGS data sets that were examined. (**G**) Bar chart showing the number of genes from the overlap (241 gene locus IDs) between the ChIP-seq and RNA-seq (as shown previously in **E**) belonging to categories of genes encoding for proteins, known to play crucial roles in signal transduction pathways. The number for each category was assigned after conducting manual examination of the set of 241 gene locus IDs for each indicative protein category of the signaling.

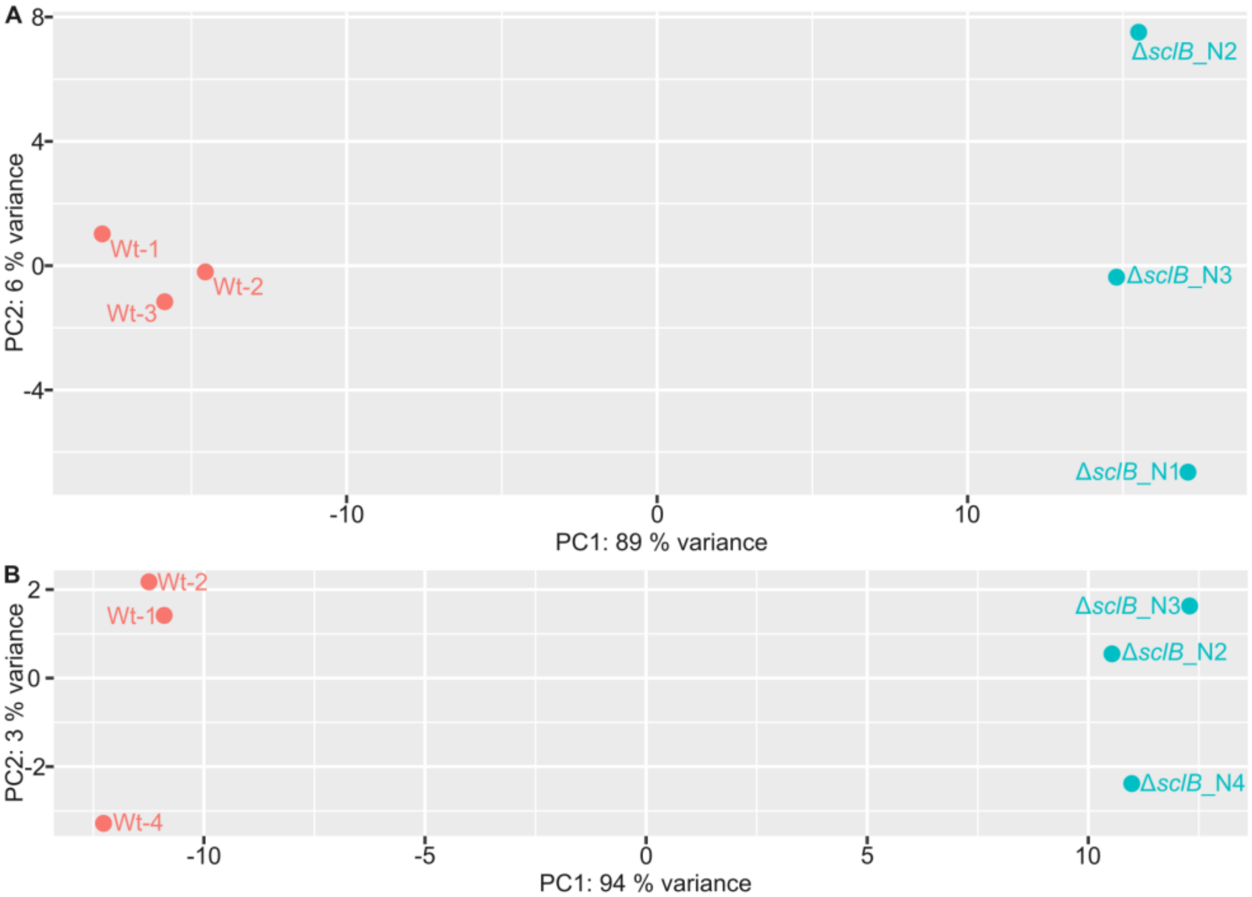

FIG S2: Clustering of different samples for the RNA-seq performed during *A. nidulans* vegetative growth or induction of asexual spore formation for Wt compared to Δ*sclB* mutant strains. Principal component analysis (PCA) among the RNA-seq samples of Wt and Δ*sclB* either grown under (**A**) Veg or (**B**) Asex conditions.

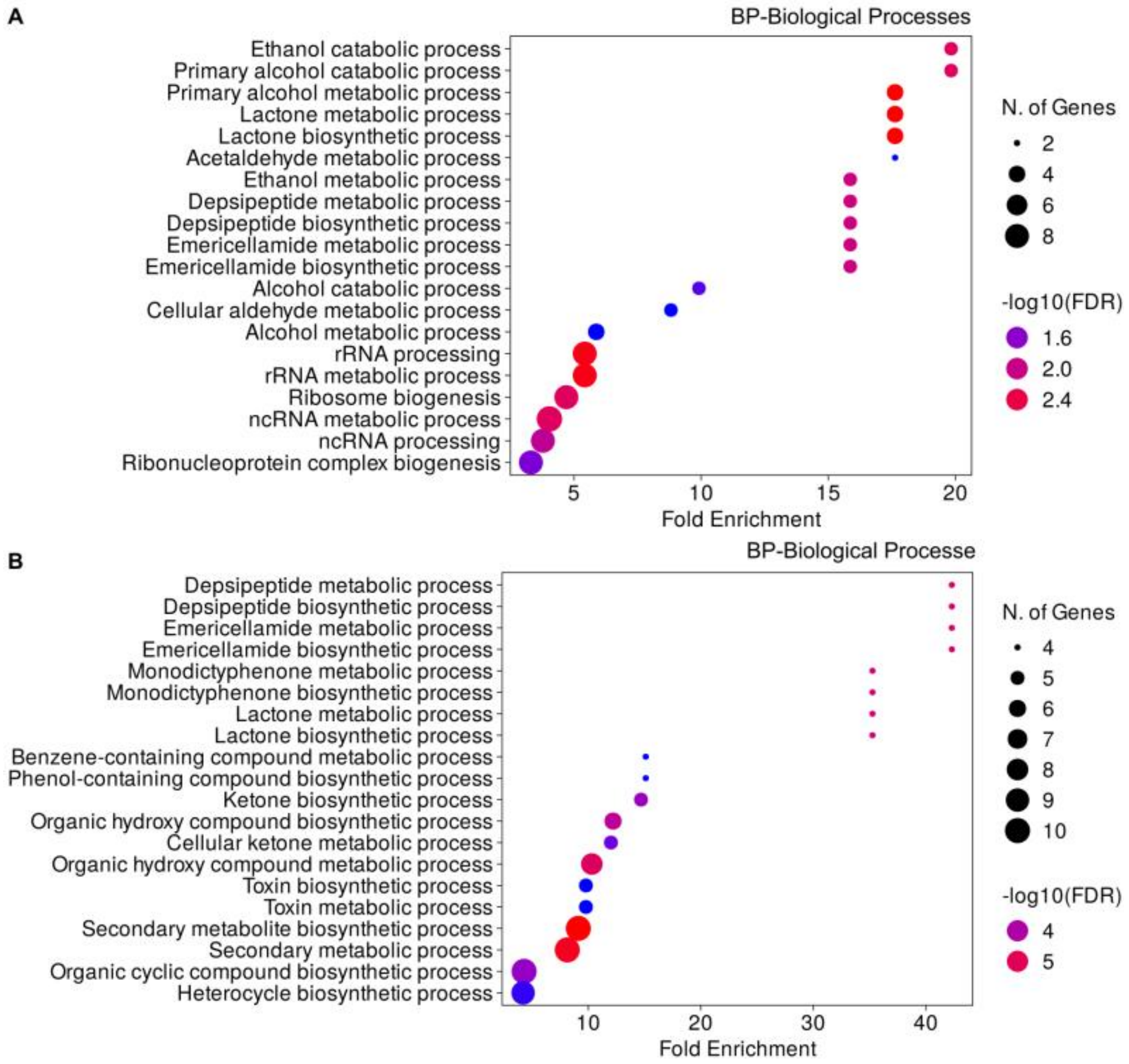

FIG S3: Proteins involved in several *A. nidulans* metabolic processes are among the top direct target genes of SclB either during vegetative growth or when asexual development is induced. Bubble plots present the Gene Ontology (GO)-enrichment analysis (in terms of biological processes/BP), of genes belonging to the overlap of ChIP-seq with the RNA-seq for mycelia either grown (**A**) under Veg (441 locus IDs) or (**B**) Asex (241 locus IDs) conditions, respectively. All IDs were submitted to cut offs for the ChIP-seq: $p < 0.05$ and fold enrichment (F.E.) ≥ 2.0 and for the RNA-seq: $p < 0.05$ and $-1 \geq \log_2 FC$(Fold Change) ≥ 1, prior to the enrichment analysis. The coloring of the bubbles corresponds to statistical differences presented as -log10(FDR). The size of the black circles reflects the size of gene sets per different identified GO-categories of biological process (BP), with which the GO-enrichment analysis was performed.

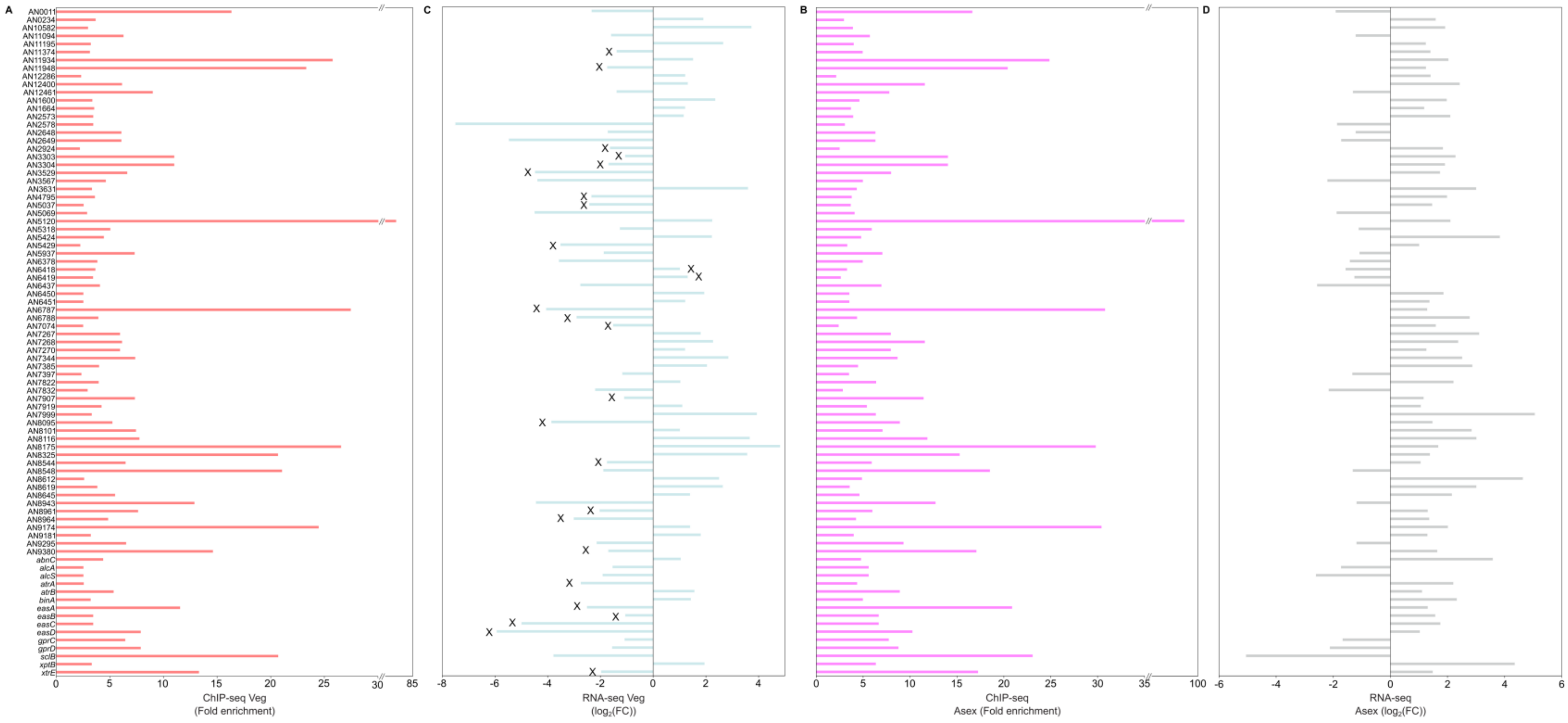
A
C
B
D
AN0011
AN0234
AN10582
AN11094
AN11195
AN11374
AN11934
AN11948
AN12286
AN12400
AN12461
AN1600
AN1664
AN2573
AN2578
AN2648
AN2649
AN2924
AN3303
AN3304
AN3529
AN3567
AN3631
AN4795
AN5037
AN5069
AN5120
AN5318
AN5424
AN5429
AN5937
AN6378
AN6418
AN6419
AN6437
AN6450
AN6451
AN6787
AN6788
AN7074
AN7267
AN7268
AN7270
AN7344
AN7385
AN7397
AN7822
AN7832
AN7907
AN7919
AN7999
AN8095
AN8101
AN8116
AN8175
AN8325
AN8544
AN8548
AN8612
AN8619
AN8645
AN8943
AN8961
AN8964
AN9174
AN9181
AN9295
AN9380
abnC
alcA
alcS
atrA
atrB
binA
easA
easB
easC
easD
gprC
gprD
sclB
xptB
xtrE
ChIP-seq Veg
(Fold enrichment)
RNA-seq Veg
(log_2(FC))
ChIP-seq
Asex (Fold enrichment)
RNA-seq
Asex (log_2(FC))

FIG S4: Specific set of common SclB controlled genes during vegetative growth or asexual development of *A. nidulans*. Bar diagrams illustrate (**A** and **C**) the ChIP-seq results (fold enrichment) and (**B** and **D**) the RNA-seq results (log_2FC) from mycelia grown under Veg or Asex conditions. Each diagram presents the corresponding values for each of the 83 gene locus IDs that were found to be simultaneous targets of GFP-SclB (in both ChIP-seqs data sets) and differentially expressed (in both RNA-seqs data sets), independently from the growth conditions. The applied thresholds for ChIP-seq were: $p < 0.05$ and fold enrichment (F.E.) ≥ 2.0, the peak must be located up to 3 kb upstream from the TSS of every gene locus; for RNA-seq: $p < 0.05$ and $-1 \geq log_2FC$(Fold Change) ≥ 1. The letter X in (**C**) bar plot, indicates the genes that show a contrary expression pattern compared to the one during the Asex growth (**D**).

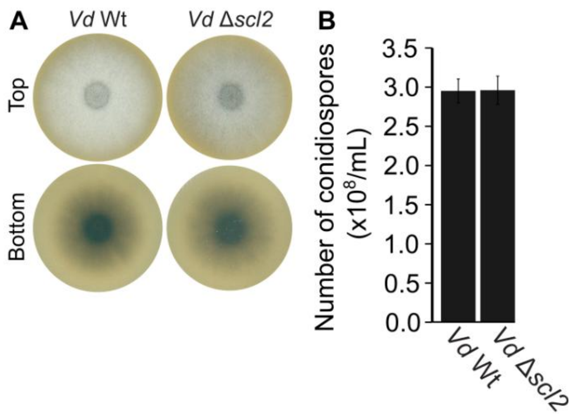

FIG S5: *V. dahliae* Scl2 is dispensable for colony formation and conidiation. (**A**) Colony growth of the *V. dahliae* Wt-JR2 in comparison to the *scl2* deletion strain (*Vd* Δ*scl2*) on pectin-rich simulated xylem medium (SXM). (**B**) Quantification of conidiospores produced by either the Wt or *Vd* Δ*scl2* in liquid SXM culture after 5 days of incubation. Bars represent the mean values of two independent experiments with 3-4 repetitions each.

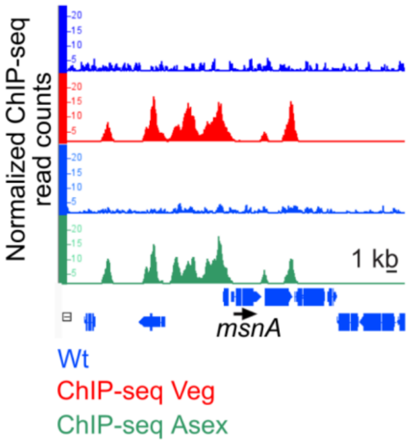

FIG S6: SclB is associated with multiple sites of the *msnA* promoter *in vivo*. The snapshot from the IGB shows the *in vivo* binding of SclB (ChIP-seq peaks) on the promoter of the *msnA* gene. Peaks appearing in the red tracks refer to the ChIP-seq performed under Veg conditions and peaks shown in green tracks under Asex conditions correspondingly. Blue tracks, refer to negative controls (background signal) of each ChIP-seq in each case.

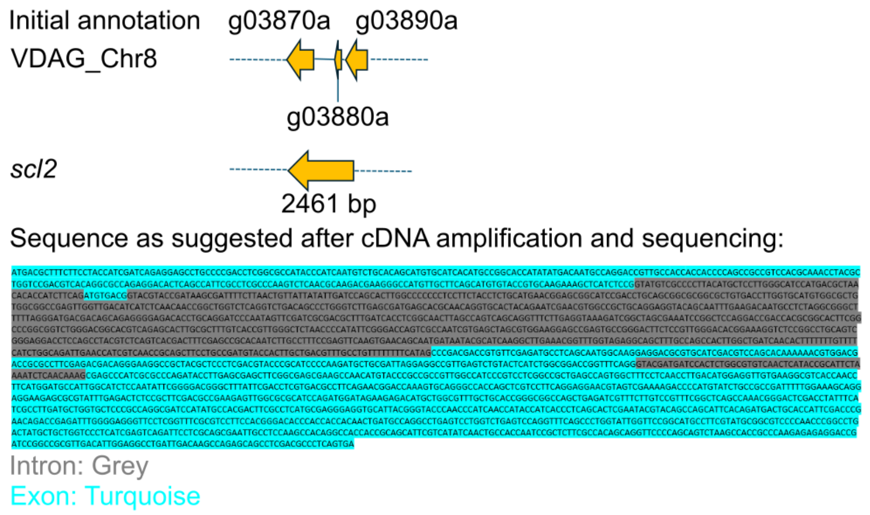

FIG S7: Prediction and annotation of *V. dahliae scl2*.
